# FMLP- and TNF-stimulated monoclonal Lym-1 antibody-dependent lysis of B lymphoblastoid tumour targets by neutrophils

**DOI:** 10.1038/sj.bjc.6690359

**Published:** 1999-05-01

**Authors:** L Ottonello, P Morone, M Mancini, M Amelotti, P Dapino, F Dallegri

**Affiliations:** Department of Internal Medicine, Semeiotica Medica 2, University of Genova Medical School, Viale Benedetto XV, n.6, I-16132 Genova, Italy

**Keywords:** neutrophils, lymphoma, immunotherapy, ADCC, TNF-α

## Abstract

Human neutrophils, incubated with Cr^51^-labelled B lymphoblastoid Raji cells in the presence of the anti-target monoclonal antibody (mAb) Lym-1 plus formyl-methionyl-leucyl-phenylalanine (FMLP) or tumour necrosis factor alpha (TNF-α), were found to induce significant Cr^51^ release, i.e. significant cytolysis. The lytic process was inhibited by mAb IV.3, specific for the Fcγ receptor (FcγR) type II. The mAb 3G8, which reacts with FcγR type III, was ineffective. Moreover, the lysis was inhibited by the anti-CD18 mAb MEM-48. These data suggest that FMLP/Lym-1 as well as TNF-α/Lym-1 cytolytic systems strictly require FcγRII and CD18 integrins. As the lysis induced by TNF-α/Lym-1 was prevented by pertussis toxin (PT), PT-sensitive G-proteins are likely to intervene in post-FcγRII signal transduction. Both the FMLP- and the TNF-α-dependent systems were also found to be equally susceptible to inhibition by various inhibitors of kinases (genistein, staurosporin, 1-(5-isoquinolinnylsulphonyl)-2-methylpiperazine and wortmannin). On the contrary, an inhibitor of protein kinase C (bis-indolyl-maleimide, BIM) was effective only in the FMLP/Lym-1 cytolytic system. Therefore, it appears that signals delivered by FMLP or TNF-α, BIM-sensitive and insensitive respectively, converge and synergize with those from G-protein-coupled FcγRII and, probably, CD18-integrins to promote the expression of the neutrophil cytolytic potential. © 1999 Cancer Research Campaign


*British Journal of Cancer* (1999) **80**(3/4), 331–337
© 1999 Cancer Research Campaign
Article no. bjoc.1998.0359

### FMLP- and TNF-stimulated monoclonal Lym-1 antibody-dependent lysis of B lymphoblastoid tumour targets by neutrophils

**L Ottonello, P Morone, M Mancini, M Amelotti, P Dapino and F Dallegri**

Department of Internal Medicine, Semeiotica Medica 2, University of Genova Medical School, Viale Benedetto XV, n.6, I-16132 Genova, Italy

**Summary** Human neutrophils, incubated with $Cr^{51}$-labelled B lymphoblastoid Raji cells in the presence of the anti-target monoclonal antibody (mAb) Lym-1 plus formyl-methionyl-leucyl-phenylalanine (FMLP) or tumour necrosis factor alpha (TNF-α), were found to induce significant $Cr^{51}$ release, i.e. significant cytolysis. The lytic process was inhibited by mAb IV.3, specific for the Fcγ receptor (FcγR) type II. The mAb 3G8, which reacts with FcγR type III, was ineffective. Moreover, the lysis was inhibited by the anti-CD18 mAb MEM-48. These data suggest that FMLP/Lym-1 as well as TNF-α/Lym-1 cytolytic systems strictly require FcγRII and CD18 integrins. As the lysis induced by TNF-α/Lym-1 was prevented by pertussis toxin (PT), PT-sensitive G-proteins are likely to intervene in post-FcγRII signal transduction. Both the FMLP- and the TNF-α-dependent systems were also found to be equally susceptible to inhibition by various inhibitors of kinases (genistein, staurosporin, 1-(5-isoquinolinnylsulphonyl)-2-methylpiperazine and wortmannin). On the contrary, an inhibitor of protein kinase C (bis-indolyl-maleimide, BIM) was effective only in the FMLP/Lym-1 cytolytic system. Therefore, it appears that signals delivered by FMLP or TNF-α, BIM-sensitive and insensitive respectively, converge and synergize with those from G-protein-coupled FcγRII and, probably, CD18-integrins to promote the expression of the neutrophil cytolytic potential.

**Keywords:** neutrophils; lymphoma; immunotherapy; ADCC; TNF-α

The ability of normal neutrophils to exert antibody-dependent cellular cytotoxicity (ADCC) towards certain human tumour cells is well-documented (Gale and Zighelboim, 1974; Clark and Klebanoff, 1977; Levy et al, 1979). In particular, using heterologous polyclonal anti-target antibodies, neutrophils have been indeed shown to lyse human B lymphoma cell lines efficiently (Dallegri et at, 1984). More recently, neutrophils incubated with selected murine anti-target monoclonal antibodies (mAb) were found to mediate consistent lysis of human melanoma or neuroblastoma cell lines (Kushner and Cheung, 1989, 1991; Baldwin et al, 1993; Ranhammar et al, 1994) and minimal or no lysis of human lymphoma or leukaemia cells (Gavioli et al, 1990; Vaickus et al, 1990). Nevertheless, using a particular (Lym-1) mAb towards lymphoblastoid Raji cells employed as a model of B-lymphoma cells, certain cytokines and chemotaxins have been found to augment or promote neutrophil-mediated ADCC (Vaickus et al, 1990; Ottonello et al, 1996). In this regard, various cytokines have been also shown to be capable to activate anti-neoplastic and leucocyte-mediated reactions in various mouse models (Pickaver et al, 1972; Fady et al, 1990; Midorikama et al, 1990). In these in vivo systems, the local addition of exogenous cytokines or their release by engineered tumour cells was indeed found to result in immune responses, leading to tumour cell lysis by distinct effectors including neutrophils (Colombo et al, 1992*a*, 1992*b*).

*Received 17 July 1998*
*Revised 27 October 1998*
*Accepted 24 November 1998*

*Correspondence to:* F Dallegri, Semeiotica Medica 2, Dipartimento di Medicina Interna, Viale Benedetto XV, n.6, I-16132 Genova, Italy

In front of the aforementioned evidence for the ability of neutrophils to exert tumoricidal activity, the mechanisms underlying this function are only partially elucidated. This is particularly true as far as the mAb-dependent neutrophil-mediated ADCC is concerned. In the presence of the mAb 3F8 specific for the ganglioside GD2 antigen, neutrophils were found to lyse melanoma and neuroblastoma cell lines through a process requiring Fcγ receptor (FcγR) type II and FcγRIII as well as adhesion molecules such as CD11a-CD18 and CD11b-CD18 integrins (Kushner and Cheung, 1992). On the other hand, using glioblastoma target cells sensitized by mAb 425, FcγRII was shown to be crucial for lysis mediated by normal neutrophils (Valerius et al, 1993). The present study was planned to further investigate the receptor requirement in mAb-dependent ADCC model systems, such as the chemotaxin- or cytokine-stimulated Lym-1 mAb-dependent ADCC of Raji target cells by neutrophils. We used formyl–methionyl–leucyl–phenylalanine (FMLP) and tumour necrosis factor alpha (TNF-α) as neutrophil stimuli, whereas Lym-1 mAbs were employed to direct effector cells toward B-lymphoblastoid Raji targets.

### MATERIALS AND METHODS

### Culture medium and reagents

The following culture medium was used: RPMI-1640 (Irvine Scientific, Santa Ana, CA, USA) supplemented with 10% heat-inactivated (56°C, 45 min) fetal calf serum (FCS; HyClone Eur. Ltd, Cramlington, NE, USA) and 2 mmol $L^{-1}$ glutamine (Irvine Scientific) (RPMI–FCS). Hanks' balanced salt solution (HBSS) was from Irvine Scientific. Ficoll Hypaque was purchased from Seromed (Berlin, Germany). Sodium chromate $Cr^{51}$ was from the

Radiochemical Centre (Amersham, UK). Bis-indolyl-meleimide (BIM) was from Calbiochem (La Jolla, CA, USA). Triton X-100, ethidium bromide, *N-formyl-met-leu-phe* (FMLP), genistein (GST), wortmannin (WMN), staurosporine (STP), 1-(5-Isoquinolinylsulphonyl)-2-methylpiperazine (H7) and bovine serum albumin (BSA) were purchased from Sigma Chemical Co. (St Louis, MO, USA). Heparin was obtained from Roche (Milan, Italy). Polyclonal human IgG were from Sclavo (Siena, Italy). Human recombinant TNF-α was purchased from BioSource International (Camarillo, CA, USA).

### Monoclonal antibodies

The previously described (Epstein et al, 1987) mAb Lym-1 (IgG2a) was used as anti-target mAb for the cytolytic assay. Moreover, the following mAbs were used: anti-CD32 IV.3 (IgG2a, Fab fragments, Medarex, Annandale, NJ, USA), anti-CD16 3G8 (IgG1, native mAb and $F(ab')_2$ fragments, Medarex), anti-CD64 197 (IgG2a, Medarex), anti-CD18 MHM23 (Ig1, Dako AS, Glostrup, Denmark), anti-CD18 MEM48 (IgG1, kindly provided by V Horejsi, Praha), anti-CD18 60.3 (IgG2a, kindly provided by J Harlan, Washington), anti-CD11a MEM25 (IgG1, kindly provided by V Horejsi, Praha), anti-CD11b 2LPM19c (IgG1, Dako AS), anti-CD11b 44 (IgG1, Biosource, Camarillo, CA, USA), anti-CD11b CBRM 1/5 (IgG1, kindly provided by TA Springer, Boston), anti-CD11c 3.9 (IgG1, Biosource), anti-CD11c KB90 (IgG1, Dako AS), anti-ICAM1 84H10 (IgG1, Immunotech, Marseille, France), anti-CD16 FITC-conjugated mAb 3G8 (IgG1, Pharmingen, San Diego, CA), anti-CD32 fluorescein isothiocyanate (FITC)-conjugated mAb FLI8.26 (IgG2b, Pharmingen), anti-CD64 FITC-conjugated mAb 10.1 (IgG1, Pharmingen), appropriate mouse IgG FITC-conjugated isotype controls 107.3 and 49.2 (Pharmingen).

### Neutrophil preparation

Heparinized venous blood (heparin 10 U $ml^{-1}$) was obtained from healthy volunteers (20–45 years old) after informed consent. No donor had an infectious disease or was under medication either at the time of sampling or for 2 weeks before sampling. Neutrophils were prepared by dextran sedimentation, followed by centrifugation (400 ***g***, 30 min) on Ficoll-Hypaque density gradient, as previously described (Ottonello et al, 1996). Contaminating erythrocytes were removed by hypotonic lysis (Ottonello et al, 1996). PMNs resuspended in RPMI–FCS were > 97% pure viable, as determined by the assays described above.

### Target cells

Lymphoblastoid Raji cells (Ottonello et al, 1996) were used as targets in the cytolytic assays. The Raji cell line was grown in RPMI–FCS and subcultured every 3 days. The capacity of these cells to bind Lym-1 antibody was measured by indirect immunofluorescence with flow cytometry using a rabbit anti-mouse IgG $F(ab')_2$ polyclonal antibody conjugated with FITC (Dako) (Ottonello et al, 1996). For cytolytic assays, $4 \times 10^6$ Raji cells were labelled with 100–200 μCi sodium chromate $Cr^{51}$ by incubating for 1 h at 37°C (final volume 0.5 ml, medium: RPMI-1640 plus 5% FCS). After washing, labelled cells were resuspended in RPMI–FCS.

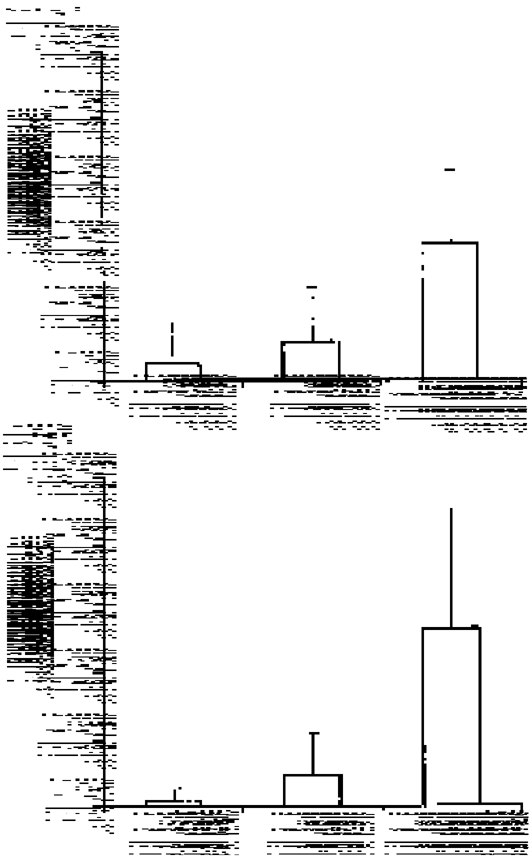

**Figure 1** Neutrophil-mediated cytolysis in the absence or presence of 10 μg $ml^{-1}$ Lym-1 and/or 1 μM FMLP or 1 ng $ml^{-1}$ TNF-α. $Cr^{51}$-labelled Raji target cells were at $2 \times 10^4$. The neutrophil:Raji cell ratio was 20:1. The incubation time was 14 h. **(A)** The lysis in the presence of both Lym-1 and FMLP was significantly higher than that in the presence of FMLP or that in the presence of Lym-1 alone, $P < 0.001$. **(B)** The lysis in the presence of both Lym-1 and TNF-α was significantly higher than that in the presence of TNF-α or that in the presence of Lym-1 alone, $P < 0.001$

### Cytolytic assays

Cytolytic activity of neutrophils was measured as described elsewhere in detail (Dallegri et al, 1984; Ottonello et al, 1996). Briefly, target cells ($2 \times 10^4$) were mixed with neutrophils at an effector:target ratio of 20:1, with and without 10 μg $ml^{-1}$ Lym-1 mAb (Epstein et al, 1987) and/or 1 μM FMLP or 1 ng $ml^{-1}$ TNF-α appropriately diluted in RPMI–FCS. The effector:target ratio of 20:1 was chosen on the basis of preliminary experiments, also taking into account previous observations (Ottonello et al, 1996). In fact, the per cent cytolysis at effector:target ratios of 20:1 and 40:1 was 28.33 ± 6.43 and 32.5 ± 3.08 (mean ± 1 standard deviation (s.d.), $n = 3$, $P > 0.05$) in the TNF-α system, whereas it was 23.97 ± 10.82 and 26.87 ± 7.34 (mean ± 1 s.d. $n = 3$, $P > 0.05$) in the FMLP system. Experiments were carried out in the absence or presence of the various mAbs and reagents used to probe the cytolytic process. The assays were carried out in triplicate and in a final volume of 150 μl, using round-bottom microplates (Falcon, Becton-Dickinson Italia, Milano, Italy). After 14-h incubation in humidified atmosphere of 95% air and carbon dioxide, the $Cr^{51}$-release was determined in the formula $100 \times (E\text{-}S)/(T\text{-}S)$, where E is the cpm released in the presence of effector cells, T is the cpm released after target cells with 5% Triton X-100, and S is the cpm

© Cancer Research Campaign 1999

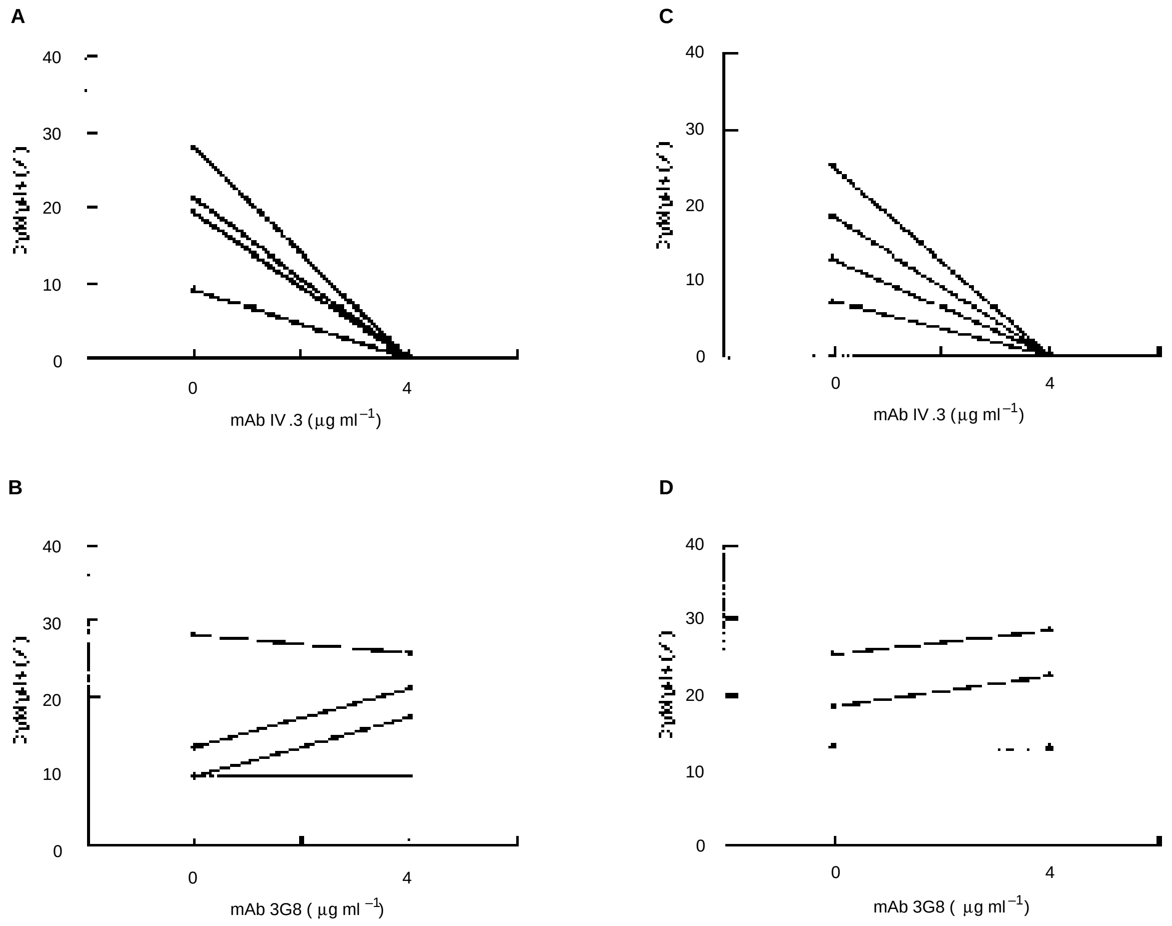

**Figure 2** Effect of the anti-CD32 (FcγRII) mAb IV.3 Fab fragments and anti-CD16 (FcγRIII) mAb 3G8 F(ab´)$_2$ fragments on FMLP- and TNF-α-stimulated neutrophil-mediated Lym-1 antibody-dependent cytolysis. $Cr^{51}$-labelled Raji cells were at 2 × $10^4$. The neutrophil: Raji cell ratio was 20:1. The incubation time was 14 h. FMLP-stimulated lysis: **(A)** cytolysis in the absence versus that in the presence of 4 µg $ml^{-1}$ IV.3: $P = 0.0286$. **(B)** Cytolysis in the absence versus that in the presence of 4 µg $ml^{-1}$ 3G8: $P = 0.685$. TNF-α-stimulated lysis: **(C)** cytolysis in the absence versus that in the presence of 4 µg $ml^{-1}$ IV.3: $P = 0.0286$. **(D)** Cytolysis in the absence versus that in the presence of 4 µg $ml^{-1}$ 3G8: $P = 1.000$

spontaneously released by target cells incubated with medium alone (< 18%).

### Immunofluorescence analysis

Neutrophils ($10^6$ cells) were incubated for 30 min at 4°C in the presence of FITC-labelled mAbs towards FcγRI, FcγRII, FcγRIII or control mAbs. Polyclonal human IgG (4 mg $ml^{-1}$) was added during incubation to inhibit possible non-specific binding of mAbs to high affinity FcγR for IgG. After incubation, the cells were washed in phosphate-buffered saline (PBS) plus 1% BSA and resuspended in PBS for analysis on a Coulter flow cytometer. To compare results, relative fluorescence intensities (RFI) were calculated as the ratios between the linear fluorescence intensity (FI) obtained with the relevant mAb and the FI obtained with the control mAb.

### Statistical analysis

Results were expressed as mean ± 1 s.d. and/or a median with the 95% confidence interval (CI). Statistical differences were analysed by the Mann–Whitney test. Significance was accepted when $P < 0.05$.

### RESULTS

### Intervention of neutrophil FcγR and β$_2$-integrins in Lym-1 mAb-dependent FMLP- and TNF-α stimulated lysis

When incubated with $Cr^{51}$-labelled Raji target cells, human neutrophils failed to cause lysis (per cent $Cr^{51}$ release: 0.25 ± 0.77, mean ± 1 s.d., $n$ = 21 with a median of 0.00 and a 95% CI from –0.10 to 0.60). As shown in Figure 1, neither FMLP nor TNF-α activated neutrophil lytic activity whereas the anti-target mAb Lym-1 caused low but significant levels of lysis. The simultaneous addition of Lym-1 and FMLP, or Lym-1 and TNF, to the neutrophil-target cell co-cultures resulted in consistent amplification of the lysis (Figure 1). In the presence of both FMLP and Lym-1, the lysis was 20.79 ± 10.87 (mean ± 1 s.d., $n$ = 77) with a median of 20.60 (95% CI, 18.32–23.26). Moreover, in the presence of both TNF-α and Lym-1, the lysis was 26.83 ± 18.36 (mean ± 1 s.d., $n$ = 39) with a median of 21.80 (95% CI, 20.87–32.78). These data suggest that FMLP and TNF-α synergistically cooperate with the anti-target mAb Lym-1 to stimulate neutrophil cytolytic activity. As shown in Figure 2, the anti-FcγRII mAb IV.3 Fab fragments (anti-CD32) inhibited the lysis of target cells by neutrophils incubated with Lym-1 and FMLP. On the other hand, Lym-1/FMLP-dependent

© Cancer Research Campaign 1999

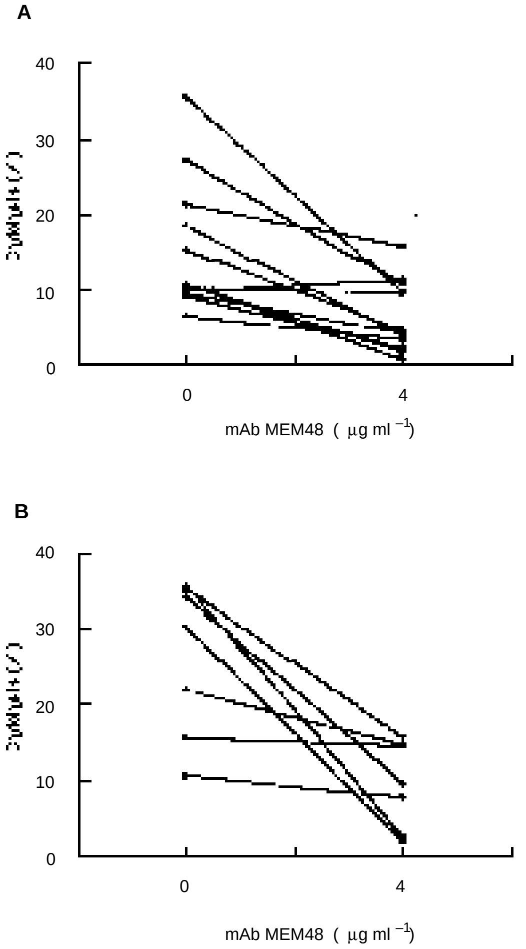

**Figure 3** Effect of the anti-CD18 mAb MEM48 on the FMLP- and TNF-α-stimulated neutrophil-mediated Lym-1 antibody-dependent cytolysis. $Cr^{51}$-labelled Raji cells were at $2 \times 10^4$. The neutrophil:Raji cell ratio was 20:1. The incubation time was 14 h. **(A)** FMLP-stimulated lysis: cytolysis in the absence versus that in the presence of MEM48: $P = 0.004$. **(B)** Cytolysis in the absence versus that in the presence of MEM48: $P = 0.004$

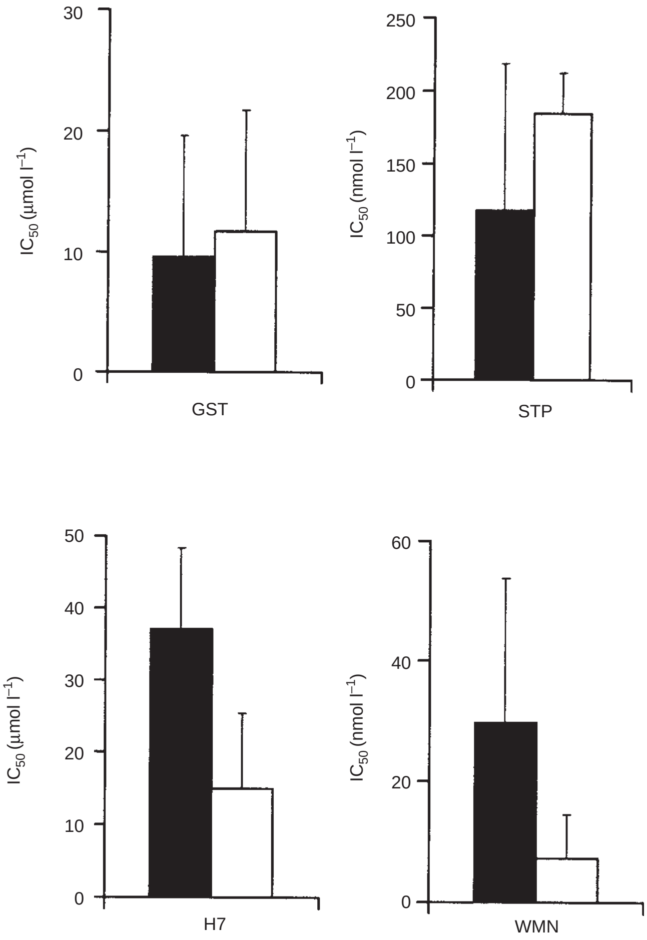

**Figure 4** Effect of genistein (GST), staurosporin (STP), 1-(5-Isoquinolilylsulphonyl)-2-methylpiperazine (H7) and wortmannin (WMN) on the FMLP- and TNF-α-stimulated Lym-1 antibody-dependent lysis. $Cr^{51}$-labelled Raji cells were at $2 \times 10^4$. The neutrophil:Raji cell ratio was 20:1. Incubation time was 14 h. Black bars show the results with FMLP and the white bars those with TNF-α. Results are expressed as mean ± 1 s.d. of the inhibitory dose 50% ($IC_{50}$). The numbers of experiments were five for GST and STP and three for H7 and WMN. For each substance, cytolysis in the presence of FMLP versus that in the presence of TNF-α: $P > 0.05$

cytolysis was unaffected by the anti-FcγRIII mAb 3G8 $F(ab')_2$ fragments (Figure 2). Similar results were obtained by using these anti-FcγR mAbs to investigate Lym-1/TNF-α-dependent lysis (Figure 2). Moreover, it is of note that native 3G8 mAb inhibited both TNF-α (26.24 ± 10.30 vs 3.1 ± 4.1, mean ± 1 s.d., $n = 7$, $P = 0.0006$) and FMLP system (20.32 ± 9.33 vs 3.74 ± 2.92, mean ± 1 s.d., $n = 5$, $P = 0.0079$). Finally, a panel of mAbs against various members of $\beta_2$-integrins were tested (data not shown), but only the anti-CD18 mAb MEM 48 inhibited the Lym-1/FMLP- and Lym-1/TNF-α-stimulated neutrophil-mediated lysis (Figure 3). Therefore, FcγRII and $\beta_2$-integrins are strictly and equally required in both the FMLP- and TNF-α-dependent cytolytic model systems. Finally, mAb 197 specific for FcγRI (CD64) did not affect neutrophil-mediated lysis (FMLP system: 27.47 ± 7.03 and 29.60 ± 7.60 in the absence and presence of 4 μg $ml^{-1}$ mAb 197; TNF-α system: 26.07 ± 5.02 and 33.0 ± 4.39 in the absence and presence of 4 μg $ml^{-1}$ mAb 197, mean ± 1 s.d., $n = 3$).

### Effect of inhibitors of distinct signalling pathways on neutrophil ADCC activity stimulated by FMLP and TNF-α

In order to understand if different post-receptor signal transduction pathways underlie neutrophil cytolytic activity in the two model systems, i.e. the Lym-1/TNF-α vs the Lym-1/FMLP system, the effect of various inhibitors was studied. GST, an inhibitor of tyrosine kinase (Rollet et al, 1994), staurosporin (STP) and H-7, which have been shown to affect the activity of various protein kinases (Ginis and Tauber, 1990) and WMN, which inhibits both phosphatidylinositol-3-kinase and phospholipase D (Vlahos et al, 1995), were found to equally suppress neutrophil activity in Lym-1/TNF-α and Lym-1/FMLP system (Figure 4). Moreover, pertussis toxin (PT, 2 μg $ml^{-1}$) was found to equally inhibit TNF-α- and FMLP-exposed neutrophils (% $Cr^{51}$ release, TNF-α system: 16.00 ± 9.47 and 2.78 ± 4.54 in the absence and presence of PT respectively, mean ± 1 s.d., $n = 6$, $P = 0.0152$; FMLP

© Cancer Research Campaign 1999

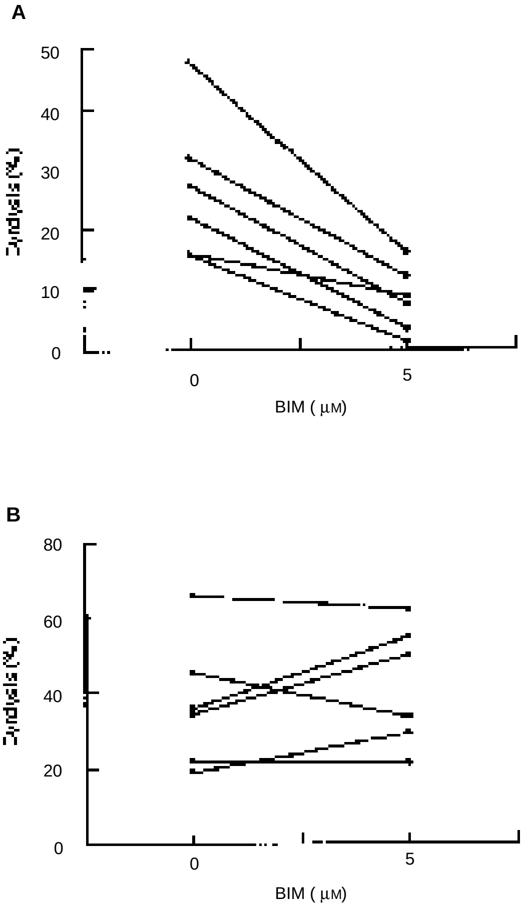

**Figure 5** Effect of bis-indolyl-maleimide (BIM, 5 μM) on the FMLP- **(A)** and TNF-α-stimulated **(B)** neutrophil-mediated Lym-1 antibody-dependent cytolysis. $Cr^{51}$-labelled Raji cells were at $2 \times 10^4$. The neutrophil:Raji cell ratio was 20:1. The incubation time was 14 h. **(A)** Cytolysis in the absence versus that in the presence of BIM: $P = 0.0087$. **(B)** Cytolysis in the absence versus that in the presence of BIM: $P = 0.008$

system: 10.73 ± 7.1 and 0.01 ± 0.04 in the absence and presence of PT respectively, mean ± 1 s.d., $n = 6$, $P = 0.0022$). On the contrary, BIM, an inhibitor of protein kinase C (Toullec et al, 1991), suppressed the lysis induced by Lym-1/FMLP-activated neutrophils without affecting the lysis induced in parallel experiments by Lym-1/TNF-α (Figure 5). The cytolytic activity of Lym-1/TNF-α-stimulated neutrophils was also unaffected by a dose of BIM ten times higher than that used in experiments reported in Figure 5 (data not shown).

### Analysis of FcγR expression on neutrophils exposed to FMLP and TNF-α

The exposure of neutrophils to 1 μM FMLP or 1 ng $ml^{-1}$ TNF-α did not significantly affect ($P > 0.05$) the levels of FcγRII expression (relative fluorescence intensities, i.e. RFI were 5.01 ± 1.36, 4.1 ± 0.8, 4.1 ± 0.3 after cell incubation for 14 h in the absence or presence of FMLP and TNF-α respectively, mean ± 1 s.d., $n = 3$). Moreover, neutrophil exposure (14 h) to FMLP or TNF-α caused a decrease in FcγRIII expression (RFI were 5.6 ± 3.9, 2.2 ± 0.5 and 2.2 ± 0.4 after cell incubation for 14 h in the absence or presence of FMLP and TNF-α respectively, mean ± 1 s.d., $n = 3$). Finally, neutrophils from healthy donors did not express FcγRI (RFI: 0.9 ± 0.1, mean ± 1 s.d., $n = 3$). Nevertheless, very low levels of FcγRI could be detected on the surface of these cells after incubation (14 h) in medium (RFI: 1.5 ± 0.2, mean ± 1 s.d., $n = 3$). Comparable levels of FcγRI expression were detected after neutrophil incubation (14 h) with FMLP or TNF-α (RFI: 1.5 ± 0.2 and 1.6 ± 0.2 for cells exposed to FMLP and TNF-α respectively, mean ± 1 s.d., $n = 3$).

### DISCUSSION

A preliminary clinical trial with Lym-1 intravenous infusion, carried out in some patients with refractory lymphoma, showed an evident reduction of lymph node size only in some cases (Hu et al, 1989). Although a number of factors can contribute to these partial responses, the inadequacy of host immune effector systems is likely to play a relevant role. In order to improve Lym-1 antibody-based therapeutic approaches, it is therefore critical to understand whether cell-mediated cytolysis can be enhanced by biological response modifiers.

The present study shows that mAb Lym-1, per se ineffective or endowed with a very low activity, interacts synergistically with FMLP or TNF to trigger neutrophil ADCC towards B lymphoblastoid tumour targets. These findings confirm and extend our initial observations (Ottonello et al, 1996). Moreover, the present data suggest that the two systems, i.e. FMLP- and TNF-α-dependent Lym-1 ADCC, share a variety of characteristics including certain receptor and post-receptor requirements. These findings may be a starting point to develop anti-tumour immune reactants, such as anti-tumour mAbs conjugated with TNF-α or FMLP to be considered for in vivo administration (Obrist et al, 1991).

The two cytolytic systems appear to be strictly dependent on the intervention of FcγRII without the involvement of FcγRIII. This directly proves the actual role of FcγRII as cytolytic trigger in neutrophil mAb-dependent ADCC. In fact, this type of receptor was identified as major trigger molecule for neutrophil ADCC by using hybridoma target cells expressing antibodies to various neutrophil surface antigens (Graziano and Fanger, 1987; Elsässer et al, 1996). In this system, neutrophils were indeed able to lyse hybridoma cells expressing antibodies to FcγRII but not those bearing mAb specific for FcγRIII (Graziano and Fanger, 1987; Elsässer et al, 1996). Consistent with our findings, FcγRII but not FcγRIII were found to cluster at the effector-target interface during neutrophil ADCC towards sheep erythrocytes (Petty et al, 1989). Moreover, Valerius and co-workers provided evidence for a crucial role for FcγRII in the lysis of mAb sensitized glioblastoma cells by normal neutrophils (Valerius et al, 1993), whereas Repp and co-workers described the intervention of this type of receptor in neutrophil-mediated lysis of Daudi lymphoma cells opsonized with specific rabbit anti-serum (Repp et al, 1991). On the other hand, the participation of additional cell surface molecules is required for optimal activity, as suggested by the ability shown herein of an anti-CD18 mAb to suppress Lym-1 ADCC. This indicates the intervention of $\beta_2$ integrins, generally thought to strengthen adhesion between effector and target cells. The finding is consistent with previous evidences for $\beta_2$ integrins intervention in neutrophil ADCC systems carried out with polyclonal anti-target antibodies (Anderson et al, 1984; Kohl et al, 1984). Finally, our data are in agreement with those of other authors (Kushner and Cheung, 1992) showing the requirement for CD18 integrins in neutrophil-mediated mAb-dependent lysis of tumour cells.

*© Cancer Research Campaign 1999*

Using melanoma and neuroblastoma cell lines as targets, Kushner and Cheung have shown that mAb-ADCC by neutrophils requires both FcγRII and FcγRIII (Kushner and Cheung, 1989, 1992). Also, other authors have proposed a role for FcγRIII in neutrophil mAb-mediated tumour lysis (Gavioli et al, 1991). No final explanation for the discrepancies about the role of FcγRIII in ADCC between these findings and our present conclusions is available. Nevertheless, it is of note that, in front of the incapacity of anti-FcγRIII F(ab´)$_2$ fragments to inhibit neutrophil Lym-1 ADCC, the same but entire anti-FcγRIII mAb (3G8) inhibited the lysis efficiently. This is consistent with the recently shown ability of 3G8 mAb to block the ligand-binding site of FcγRII with its Fc portion (Flesch et al, 1997). Therefore, the inhibition of ADCC by 3G8 mAb observed by Kushner and Cheung (1989, 1992) might reflect the blockade of FcγRII. Similarly, neutrophil-mediated FcγRII-dependent phagocytosis was found to be susceptible to inhibition by native mAb 3G8 (Flesch et al, 1997). On the other hand, it is known that chemoattractant-stimulated neutrophils undergo shedding of FcγRIII (Huizinga et al, 1990; Tosi and Zakem, 1992), a phenomenon balanced at least in part by a concomitant translocation of receptors from intracellular storage compartments (Tosi and Zachem, 1992). In substantial agreement with these studies, the prolonged exposure to FMLP or TNF-α resulted in a partial down-regulation of the FcγRIII expression. Nevertheless, owing to the considerable levels of FcγRIII expression even after 14 h incubation with FMLP and TNF-α, i.e. ~ 40% of the values detectable on cells incubated in medium alone, it seems unlikely that a stimulus-induced loss of FcγRIII can account for the herein found inability of this receptor to play a role in Lym-1 ADCC.

It has been shown that FcγRI, inducible in neutrophils by interferon gamma (IFN-γ) and granulocyte colony-stimulating factor (G-CSF) (Buckle and Hogg, 1989; Repp et al, 1991; Elsässer et al, 1996; Valerius et al, 1997) is effective in activating neutrophil lytic potential, as demonstrated in reverse cytotoxic assays against anti-FcγRI mAb-producing hybridoma targets (Elsässer et al, 1996) and in tumour cell lysis mediated by bi-specific mAbs with one specificity for FcγRI (Elsässer et al, 1996; Valerius et al, 1997; Würflein et al, 1998). In agreement with other authors (Buckle and Hogg, 1989), neutrophils from healthy donors did not express FcγRI. This receptor was, however, detected after 14-h incubation, although the amount was very low, confirming previous findings (Buckle and Hogg, 1989). Nevertheless, neither FMLP nor TNF-α were found to affect neutrophil FcγRI expression and, on the other hand, neither FMLP- nor TNF-α-stimulated Lym-1 ADCC were inhibited by the anti-FcγRI mAb 197. Therefore, this Fc receptor has no role under our conditions. This consistent with the known inability of normal neutrophils to lyse anti-FcγRI mAb-expressing hybridoma cells (Elsässer et al, 1996).

Since FMLP and TNF-α were herein found to be devoid of effects on FcγRII expression but capable of triggering FcγRII-mediated ADCC, the present data are consistent with the possibility that both FMLP and TNF-α act on post-Fc receptor signal transduction systems. It is well-known that post-receptor intracellular signalling pathways leading to specific neutrophil functional responses involve various kinases, phospholipases, calcium and certain signal-transducing proteins such as, for instance, G-proteins (Nishizuka, 1995). The inhibitory activity of pertussis toxin in the FMLP–ADCC system can conceivably involve inhibition of toxin-sensitive and FMLP receptor-coupled G-proteins (Snyderman and Uhing, 1992). As neither TNF-α-dependent cell stimulation nor $\beta_2$-integrin signalling are known to involve pertussis toxin-sensitive pathways (Dinarello, 1992; Hynes, 1992), the inhibitory activity of the toxin in the TNF-α-dependent ADCC system might be attributed to the blockade of the pertussin toxin-sensitive src-like tyrosine kinase fgr pathway which has been previously shown to be associated with the FcγRII signal transduction (Hamada et al, 1993; Zhou et al, 1995). This FcγR has been indeed shown to initiate transmembrane signals that can involve pertussis toxin-sensitive pathways (Gresham et al, 1987; Feister et al, 1988). On the other hand, the protein kinase C inhibitor BIM was found to suppress FMLP- but not TNF-α-dependent ADCC, suggesting that it selectively interferes with FMLP signal transduction. On the contrary, other chemicals, including inhibitors of tyrosine kinase and phosphatidylinositol-3-kinase were equally effective in the FMLP- and TNF-α-system, suggesting that the two ADCC conditions share common activating circuits.

In conclusion, taking into account the present observations, Lym-1 ADCC can be envisaged as a process involving the coordinated intervention of various neutrophil receptors. In other terms, signals delivered by FMLP or TNF, BIM-sensitive and insensitive respectively, converge and synergize with those from G-protein coupled FcγRII and presumably $\beta_2$-integrins to induce the activation and expression of the neutrophil cytolytic potential.

### ACKNOWLEDGEMENTS

Lym-1 monoclonal antibody was kindly provided by Prof Alan L Epstein (Department of Pathology, University of Southern California, Los Angeles, CA, USA). This work has been supported by a grant (Progetto di Ricerca di Ateneo 1997) from University of Genova to FD.

### REFERENCES

Anderson DC, Schmalstieg FC, Arnaout MA, Kohl S, Tosi MF, Dana N, Buffone GJ, Hughes BJ, Brinkely BR, Dickey WD, Abramson JS, Springer T, Boxer LA, Hollers JM and Smith CW (1984) Abnormalities of polymorphonuclear leukocyte function with a heritable deficiency of high molecular weight surface glycoproteins (GP138): common relationship to dimished cell adherence. *J Clin Invest* **74**: 536–551

Baldwin GC, Chung GY, Kaslander C, Esmail T, Reinsfeld RA and Golde DW (1993) Colony-stimulating factor enhancement of myeloid effector cell cytotoxicity toward neuroectodermal tumour cells. *Br J Haematol* **83**: 545–553

Buckle AM and Hogg N (1989) The effect of IFN-γ and colony-stimulating factors on the expression of neutrophil cell membrane receptors. *J Immunol* **143**: 2295–2301

Clark RA and Klebanoff SJ (1977) Studies on the mechanism of antibody-dependent polymorphonuclear leukocyte-mediated cytoxicity. *J Immunol* **119**: 1413–1418

Colombo MP, Lombardi L, Stoppacciaro A, Melani C, Parenza M, Bottazzi B and Parmiani G (1992*a*) Granulocyte-colony stimulating factor (G-CSF) gene transduction in murine adenocarcinoma drives neutrophil-mediated tumor inhibition in vivo. *J Immunol* **145**: 113–119

Colombo MP, Modesti A, Parmiani G, Forni G (1992*b*) Local cytokine availability elicits tumor rejection and systemic immunity through granulocyte-T-lymphocyte cross-talk. *Cancer Res* **52**: 4853–4857

Dallegri F, Patrone F, Frumento G and Sacchetti C (1984) Antibody-dependent killing of tumor cells by polymorphonuclear leukocytes. Involvement of oxidative and nonoxidative mechanisms. *J Natl Cancer Inst* **73**: 331–339

Dinarello CA (1992) Role of interleukin-1 and tumor necrosis factor in systemic responses to infection and inflammation. In *Inflammation: Basic Principles and Clinical Correlates*, Gallin JI, Goldstein IM and Snyderman R (eds), pp. 211–232. Raven Press Ltd: New York

Elsässer D, Valerius T, Repp R, Weiner GJ, Deo Y, Kalden JR, van de Winkel JGJ, Stevenson GT, Glennie MJ and Gramatzki M (1996) HLA class II as potential

 © Cancer Research Campaign 1999

target antigen on malignant B cells for therapy with bispecific antibodies in combination with granulocyte colony-stimulating factor. *Blood* **87**: 3803–3812

Epstein AL, Mader RJ, Winter JN, Stathopoulos E, Chen FM, Parker JW and Taylor CR (1987) Two new monoclonal antibodies, Lym-1 and Lym-2, reactive with human B-lymphocytes and derived tumors, with immunodiagnostic and immunotherapeutic potential. *Cancer Res* **47**: 830

Fady C, Reisser D and Martin F (1990) Non-activated rat neutrophils kill syngeneic colon tumor cell by the release of low molecular weight factor. *Immunobiology* **181**: 1–12

Feister AJ, Browder B, Willis HE, Mohanakumar T and Ruddy S (1988) Pertussis toxin inhibits human neutrophil responses mediated by the 42-kilodalton IgG Fc receptor. *J Immunol* **141**: 228–233

Flesch BK, Achter G and Neppert J (1997) Inhibition of monocyte and polymorphonuclear granulocyte immune phagocytosis by monoclonal antibodies specific for FcγRI, II and III. *Ann Hematol* **74**: 15–22

Gale RP and Zighelboim J (1974) Modulation of polymorphonuclear leukocyte-mediated antibody-dependent cellular cytotoxicity. *J Immunol* **113**: 1793–1800

Gavioli R, Spisani S, Giuliani AL and Traniello S (1990) Dual mechanism in induction of human neutrophil cytotoxicity: activation of protein kinase C and elevation in intracellular calcium. *Clin Exp Immunol* **80**: 247–251

Gavioli R, Spisani S, Giuliani AL, Cosulich E, Risso A and Traniello S (1991) CD16 and C3 receptors distinguish between the two mechanisms of tumour cytotoxicity in neutrophils. *Br J Haematol* **79**: 170–176

Ginis I and Tauber AI (1990) Activation mechanisms of adherent human neutrophils. *Blood* **76**: 1233–1239

Graziano RF and Fanger MW (1987) FcγRI and FcγRII on monocytes and granulocytes are cytotoxic trigger molecules for tumor cells. *J Immunol* **139**: 3536–3541

Gresham HD, Clement LT, Volanakis JE and Brown EJ (1987) Cholera toxin and pertussis toxin regulate the Fc receptor-mediated phagocytic response of human neutrophils in a manner analogous to regulation by monoclonal 1C2. *J Immunol* **139**: 4159–4166

Hamada F, Aoki M, Akiyama T and Toyoshima K (1993) Association of immunoglobulin G Fc receptor II with Src-like protein-tyrosine kinase with Fgr in neutrophils. *Proc Natl Acad Sci USA* **90**: 6305–6309

Hu E, Epstein AL, Naeve Hu E, Epstein AL, Naeve GS, Gill I, Martin S, Sherrod A, Nichols P, Chen D, Mazumder A and Levine AM (1989) A phase 1a clinical trial of Lym-1 monoclonal antibody serotherapy in patients with refractory B cell malignancies. *Hematd Oncol* **7**: 155–166

Huizinga TWJ, de Haas M, Kleijer M, Nuijens JH, Roos D and van dem Borne AEGK (1990) Soluble Fcγ receptor III in human plasma originates from release by neutrophils. *J Clin Invest* **86**: 416–423

Hynes RO (1992) Integrins: versatility, modulation, and signaling in cell adhesion. *Cell* **69**: 11–25

Kohl S, Springer TA, Schmalstieg FC, Loo LS and Anderson DC (1984) Defective natural killer cytotoxicity and polymorphonuclear leukocyte antibody-dependent cellular cytotoxicity in patients with LFA-1/OKM-1 deficiency. *J Immunol* **133**: 2972–2978

Kushner BH and Cheung NKV (1989) GM-CSF enhances 3F8 monoclonal antibody-dependent cellular cytotoxicity against human melanoma and neuroblastoma. *Blood* **73**: 1936–1941

Kushner BH and Cheung NKV (1991) Clinically effective monoclonal antibody 3F8 mediates nonoxidative lysis of human neuroectodermal tumor cells by polymorphonuclear leukocytes. *Cancer Res* **51**: 4865–4870

Kushner BH and Cheung NKV (1992) Absolute requirement of CD11/CD18 adhesion molecules, FcRII, and the phosphatidylinositol-linked FcRIII for monoclonal antibody-mediated neutrophil antihuman tumor cytotoxicity. *Blood* **79**: 1484–1490

Levy PC, Show GM and LoBuglio AF (1979) Human monocyte, lymphocyte, and granulocyte antibody-dependent cell-mediated cytotoxicity toward tumor cells. I. General characteristic of cytolysis. *J Immunol* **123**: 594–599

Midorikama Y, Yamashita T and Sendo F (1990) Modulation of the immune response to transplanted tumors in rats by selective depletion of neutrophils *in vivo* using a monoclonal antibody: abrogation of specific transplantation resistance to chemical neutrophils in vivo. *Cancer Res* **50**: 6243–6247

Nishizuka Y (1995) Protein kinase C and lipid signalling for sustained cellular responses. *FASEB J* **9**: 484–496

Obrist R, Schmidli J, Müller R, Gallati H and Obrecht JP (1991) Acute and subacute toxicity of chemotactic conjugates between monoclonal antibody and fMet-Leu-Phe in humans: a phase I clinical trial. *Cancer Immunol Immunother* **32**: 406–408

Ottonello L, Morone P, Dapino P and Dallegri F (1996) Monoclonal Lym-1 antibody-dependent lysis of B-lymphoblastoid targets by human complement and cytokine-exposed mononuclear and neutrophilic polymorphonuclear leukocytes. *Blood* **87**: 5171–5178

Petty HR, Francis JW and Anderson CL (1989) Cell surface distribution of Fc receptors II and III on living human neutrophils before and during antibody dependent cellular cytotoxicity. *J Cell Physiol* **141**: 598–605

Pickaver AH, Ratcliffe NA, Williams AE and Smith H (1972) Cytotoxic effects of peritoneal neutrophils on a syngeneic rat tumor. *Nat New Biol* **235**: 187–189

Ranhammar P, Frödin JE, Trotta PP and Mellstedt H (1994) Cytotoxicity of white blood cells activated by granulocyte-colony-stimulating factor and macrophage colony-stimulating factor against tumor cells in the presence of various monoclonal antibodies. *Cancer Immunol Immunother* **39**: 254–262

Repp R, Valerius T, Sendler A, Gramatki M, Iro H, Kalden JR and Platzer E (1991) Neutrophils express the high affinity receptor for IgG (FcγRI, CD64) after in vivo application of recombinant human granulocyte colony-stimulating factor. *Blood* **78**: 885–889

Rollet E, Caon AC, Roberge CJ, Liao NW, Malawista SE, McColl SR and Naccache PH (1994) Tyrosine phosphorylation in activated neutrophils. Comparison of the effects of different classes of agonists and identification of the signalling pathways involved. *J Immunol* **153**: 353–363

Snyderman R and Uhing RJ (1992) Chemoattractant stimulus-response coupling. In *Inflammation: Basic Principles and Clinical Correlates*, Gallin JI, Goldstein IM and Snyderman R (eds), pp 421–439. Raven Press: New York

Tosi MF and Zackem H (1992) Surface expression of Fcγ receptor III (CD16) on chemoattractant-stimulated neutrophils is determine by both surface shedding and translocation from intracellular storage compartments. *J Clin Invest* **90**: 462–470

Toullec D, Pianetti P, Coste H, Bellevergue B, Grand-Perret T, Ajakane M, Baudet V, Boissin P, Boursier E and Loriolle F (1991) The bisindolylmaleimide GF 109203X is a potent and selective inhibitor of protein kinase C. *J Biol Chem* **266**: 15771–15781

Vaickus L, Biddle W, Cemerlic D and Foon KA (1990) Interferon gamma augments Lym-1-dependent, granulocyte-mediated tumor cell lysis. *Blood* **75**: 2408–2416

Valerius T, Repp R, de Witt TPM, Berthold S, Platzer E, Kalden JR, Gramatzki M and van de Winkel JGJ (1993) Involvement of the high-affinity receptor for IgG (FcγRI, CD64) in enhanced tumor cell cytotoxicity of neutrophils during granulocyte colony-stimulating factor therapy. *Blood* **82**: 931–939

Valerius T, Elsässer D, Repp R, van de Winkel JGJ, Gramatzki M and Glennie M (1997) HLA class II antibodies recruit G-CSF activated neutrophils for treatment for B cell malignancy. *Leukemia Lymphoma* **26**: 261–269

Vlahos CJ, Matter WF, Brown RF, Traynor-Kaplan AE, Heyworth PG, Prossnitz ER, Ye RD, Marder P, Schelm JA, Rothfuss KJ, Serlin BS and Simpson PJ (1995) Investigation of neutrophil signal transduction using specific inhibitor of phosphatidylinositol 3-kinase. *J Immunol* **154**: 2413–2422

Würflein D, Dechant M, Stockmeyer B, Tutt AL, Hu P, Repp R, Kalden JR, van de Wikel JGJ, Epstein AL, Valerius T, Glennie M and Gramatzki M (1998) Evaluating antibodies for their capacity to induce cell-mediated lysis of malignant B cells. *Cancer Res* **58**: 3051–3058

Zhou M, Lublin DM, Link DC and Brown EJ (1995) Distinct tyrosine kinase activation and triton X-100 insolubility upon FcγRII or FcγRIIIB ligation in human polymorphonuclear leukocytes. Implications for immune complex activation on the respiratory burst. *J Biol Chem* **270**: 13553–13560

© Cancer Research Campaign 1999 
